# Circadian rhythm disruption: a potential trigger in Parkinson’s disease pathogenesis

**DOI:** 10.3389/fncel.2024.1464595

**Published:** 2024-10-30

**Authors:** Ke Xu, Yu Zhang, Yue Shi, Yake Zhang, Chengguang Zhang, Tianjiao Wang, Peizhu Lv, Yan Bai, Shun Wang

**Affiliations:** ^1^The Second Clinical Medical College, Heilongjiang University of Traditional Chinese Medicine, Harbin, China; ^2^Institute of Acupuncture and Moxibustion, Heilongjiang Academy of Traditional Chinese Medicine, Harbin, China

**Keywords:** circadian rhythm, Parkinson’s disease (PD), dopamine (DA), microglia, αsynuclein (α-syn), non-motor symptom

## Abstract

Parkinson’s disease (PD) is a neurodegenerative disease characterized by the gradual loss of dopaminergic neurons in the substantia nigra pars compacta (SNpc), abnormal accumulation of α-synuclein (α-syn), and activation of microglia leading to neuroinflammation. Disturbances in circadian rhythm play a significant role in PD, with most non-motor symptoms associated with disruptions in circadian rhythm. These disturbances can be observed years before motor symptoms appear and are marked by the emergence of non-motor symptoms related to PD, such as rapid eye movement sleep behavior disorder (RBD), restless leg syndrome (RLS), excessive daytime sleepiness (EDS), depression and anxiety, changes in blood pressure, gastrointestinal dysfunction, and urinary problems. Circadian rhythm disruption precedes the onset of motor symptoms and contributes to the progression of PD. In brief, this article outlines the role of circadian rhythm disruption in triggering PD at cellular and molecular levels, as well as its clinical manifestations. It also explores how circadian rhythm research can contribute to preventing the onset and progression of PD from current and future perspectives.

## 1 Introduction

The circadian rhythm refers to a 24-hour cycle from day to night. The internal mechanism responsible for regulating the circadian rhythm is known as the “biological clock,” which consists of both central and peripheral components (Meyer et al., 2022). In particular, the suprachiasmatic nucleus (SCN) located in the hypothalamus serves as the central biological clock and acts as a “pacemaker” for maintaining this rhythm (Montaruli et al., 2021). The SCN not only regulates its own tissue-specific rhythms but also detects light and temperature signals while influencing peripheral biological clocks through endocrine signaling pathways and autonomic nerves (Speksnijder et al., 2024). These peripheral biological clocks are found in various organs such as liver, skin, heart, kidney etc., each exhibiting their own rhythmicity under central regulation (Bass, 2024).

**FIGURE 1:**
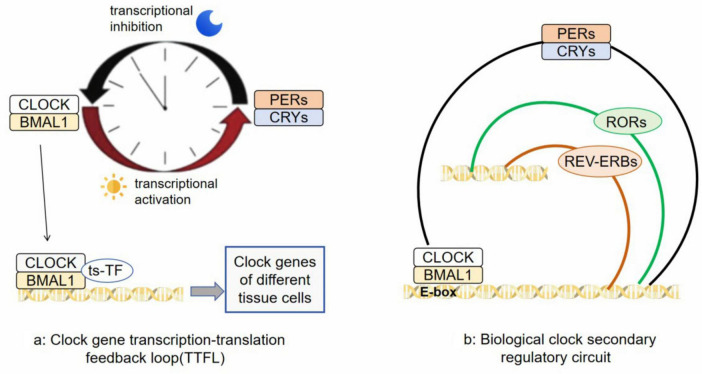
**(a)** is a transcription-translation feedback loop (TTFL) composed of CLOCK, BMAL1, PERs and CRYs; **(b)** is REV-ERBs and RORs constitute the secondary regulatory circuit of the core biological clock.

In the 1970s, a significant discovery was made by American molecular biologist Benzer S and his student Konopka R. They found that mutations in an unidentified gene in fruit flies disrupted their circadian rhythms. This gene was later named Period (PER) (Konopka and Benzer, 1971). In the 1980s, researchers successfully isolated the PER gene along with its coding protein, PER. It was observed that levels of the PER protein increase during nighttime and decrease throughout the day, following a 24-hour cycle synchronized with the circadian rhythm (James et al., 1986). After extensive research spanning several years, it was revealed that circadian homeostasis is maintained by central clock core genes located in the hypothalamic SCN through a transcription-translation feedback loop (TTFL) (Li et al., 2023). During daytime, CLOCK and BMAL1 genes form dimers and bind to E-box elements on promoter regions of clock-controlled genes (CCGs), activating expression through transcription. As transcriptional activators, CLOCK/BMAL1 also stimulate expression of suppressor gene families such as PER and CRY. At nightfall, levels of PERs (including PER1, PER2 and PER3) and CRYs (including CRY1, CRY2 and CRY3) proteins increase leading to dimerized PER/CRY being transported into the nucleus where they inhibit CLOCK/BMAL1-mediated transcription (Figure 1a; Koike et al., 2012). PER and CRY undergo various modifications after translation, leading to their degradation and initiating a fresh cycle of circadian rhythms. The CLOCK/ BMAL1 and PER/ CRY components form the TTFL system, which regulates the transcription of over 4000 CCGs. This ensures synchronization between the central clock and peripheral clocks (Zhu and Belden, 2020). REV-ERBs and RORs constitute the secondary regulatory circuit of the core biological clock (Figure 1b; Patton and Hastings, 2023). The peripheral biological clock is influenced by neuronal input from tissues and organs, hormonal activity, dietary factors, timing of meals, as well as substances like drugs or alcohol. It also possesses a certain level of autonomy (Zhang et al., 2020). TTFL is present in nearly all cells throughout the body. In different tissues, CLOCK/ BMAL1 collaborates with tissue-specific transcription factors (ts-TF) to guide gene transcription related to circadian control (Tamaru and Takamatsu, 2018). Cellular circadian rhythms are regulated through integration of autonomous TTFL mechanisms with cellular metabolism and signals from the extracellular system.

Circadian rhythm is closely related to Parkinson’s disease (PD). Transgenic mice overexpressing α-synuclein (a model of PD) show greater fragmentation and amplitude reduction in locomotor activity rhythms during the active phase, and these effects progress with age (Kudo et al., 2011). SCN neuronal firing is also reduced in these mice from young adulthood and progressively worsens with age, suggesting a weakened circadian pacemaker and a potential loss of dopaminergic modulation of circadian rhythms via midbrain, striatal and SCN circuits (Korshunov et al., 2017). PD, in particular, is strongly associated with rapid eye moment sleep behaviour disorder, as well as excessive daytime sleepiness, insomnia and restless legs syndrome. In fact, more than 80% of people with RBD are later diagnosed with PD (Barone et al., 2009).

## 2 Cellular molecular perspective: the impact of circadian rhythm disturbance on PD

### 2.1 Circadian rhythm disturbance and dopamine loss

DA serves as a significant indicator of the prodromal stage in PD. According to Ruediger Hilker and his research team, non-motor symptoms associated with the prodrome of PD manifest when there is a 31% decline in DAergic neurons within the striatum (Hilker et al., 2005). The synthesis of DA can be regulated by circadian rhythm genes. Tyrosine hydroxylase (TH), an enzyme crucial for DA production, is influenced by the CLOCK gene’s circadian movement cycle. By targeting E-Box elements located in the promoter region of these genes related to DA, it can control transcription processes involving TH, active transporters for DA, and D1 receptors (Guo et al., 2018). Clock genes have the potential to regulate both daily fluctuations in DAergic activity and even influence the functioning of our biological clock itself (Verwey et al., 2016).

BMAL1 is essential for the proper functioning of the central clock, and PD patients have been reported to exhibit decreased expression of the core clock gene BMAL1 (Li et al., 2021). In mice, loss of germ line BMAL1 leads to oxidative stress and synaptic loss in the brain, which can worsen DAergic neurodegeneration caused by the toxin 1-methyl-4-phenyl-1,2,3,6-tetrahydropyridine (MPTP) (Liu et al., 2020). It has been found that cell type-specific BMAL1 loss leads to spontaneous loss of TH and DAergic neurons in SNpc. Disruption of circadian rhythms through light exposure did not replicate this phenomenon. An unbiased analysis demonstrated a 23% reduction in TH neurons within the SNpc region of TH-BMAL1 KD mice compared to control group. These findings suggest that BMAL1, as a core component of the cellular clock machinery, autonomously influences the survival of DAergic neurons in vivo. Additionally, it is worth noting that SNpc DAergic neurons are particularly vulnerable to oxidative stress and mitochondrial dysfunction (Kanan et al., 2024). This may explain why neuron loss was observed specifically in SNpc but not in other regions such as hippocampus among BMAL-KO mice. Overall, results highlight the crucial role played by BMAL1 in regulating DAergic neuron survival at a cellular level and emphasize its potential significance for developing neuroprotective strategies against PD.

The circadian rhythm is based on the periodic oscillation of PER (Brown et al., 2019). PD models consistently show dysfunction or loss of DA neurons in the PAM and PPL1 clusters (Aryal and Lee, 2019). Using a molecular clockless Per null (Per0) mutant fruit fly carrying a white mutation, the study found that loss of the clock gene led to age-dependent loss of PAM neurons, and the study identified PAM-α1 neurons as one of the susceptible DA neuron subtypes (Majcin Dorcikova et al., 2023).

The circadian receptor REV-ERBα plays a crucial role in regulating behaviors related to emotions (Chen et al., 2023). Mice lacking REV-ERBα display manic-like behavior, characterized by decreased anxiety, a depressive phenotype, increased activity levels, heightened risk-taking tendencies, and elevated aggression. Inhibiting the activity of REV-ERBα through pharmacological means, particularly in the ventral midbrain region, induces similar behavioral patterns and promotes a state of high DA levels. REV-ERBα directly hinders the expression of TH, which is an essential enzyme for DA synthesis and competes with nuclear receptor-associated 1 (NURR1) protein. This interaction establishes the circadian rhythm of the DA system. By recruiting HDAC3 and regulating histone acetylation according to circadian rhythms, REV-ERBα inhibits TH gene expression (Kim et al., 2017). These mechanisms contribute to both the circadian nature and mood regulation within the DAergic system. Another research team similarly found that administering SR8278 (an antagonist targeting REV-ERBα) via local microinjection into the ventral tegmental area (VTA) at dawn had antidepressant and anti-anxiety effects in mice with 6-OHDA lesions. This treatment restored rhythmic mood-related behaviors. The transcription levels of REV-ERBα and Nurr1 were time-dependently altered in residual VTA DAergic neurons following 6-OHDA damage. Additionally, there was an unusual antagonistic cross-talk between these two nuclear receptors (Kim et al., 2022). Therefore, it can be concluded that REV-ERBα is a significant factor contributing to non-motor symptoms associated with mental disorders in PD.

Wianny’s study transplanted macaque neural precursor (NP) into multiple functional domains of the basal ganglia in both hemispheres of middle-aged PD macaques. Previous studies have demonstrated that successful integration of these transplants leads to significant clinical improvements and a partial restoration of circadian rhythms and cognitive function, which is unprecedented. Moreover, the bilateral multisite NP grafts were found to have neuroprotective effects on both viable DA cells (VTA and SN dorsal-medial) and their ventral striatal target cells. Interestingly, there was a notable increase in striatal DA transporter binding following the transplantation. In monkeys with MPTP-induced injury to SN-striatal DA, environmental cues masked the inability of intact SCN to regulate the striatal clock gene and control resting-wake motor rhythm associated with dopamine function. Therefore, by promoting circadian rhythm recovery, NP transplantation also facilitated dopamine recovery (Wianny et al., 2022).

The involvement of melanopsin-containing retinal ganglion cells (mRGCs) is crucial in the establishment of non-image vision pathways projected to various regions of the central nervous system, effectively mediating a wide range of physiological responses, such as circadian entrainment, acute motor control, sleep regulation, and pupillary light reflex (Ortuño-Lizarán et al., 2020). mRGCs play a dominant role in the SCN, which is responsible for synchronizing circadian rhythms. They also have a significant influence on the lateral hypothalamus and ventrolateral preoptic nucleus, both of which are involved in regulating sleep behavior. Post-mortem studies indicate that PD patients exhibit substantial degradation of the retinal melanopsin positive system, resulting in reduced density and morphological changes of mRGCs compared to individuals without PD. Furthermore, there appears to be a bidirectional relationship between the degeneration of mRGCs and the reduction of dopamine-producing cells in PD, as well as loss of synaptic connections with melanopsin cells. Evidence suggests that mRGCs are regulated by dopamine released innervatively by dopaminergic cells, thereby contributing to a sustained dopaminergic response to light (Samizadeh et al., 2023).

In addition to utilizing animal models, recent studies have incorporated mathematical models to elucidate the impact of circadian rhythm on DAergic systems (Kim et al., 2023). A mathematical model has been developed to simulate the mammalian biological clock and its downstream effects on the DAergic system. This model exhibits a nearly 24-hour rhythm influenced by both light and non-light inputs, while still retaining essential characteristics of the biological clock. The model demonstrates various dynamics such as decoupling, quasi-periodicity, and chaos. Research findings indicate that when other clock variables remain periodic, BMAL1 and BMAL1-CLOCK lose their rhythmicity leading to low-amplitude oscillations in DAergic variables (Kim and Witelski, 2022). Another research team constructed a differential equation-based mathematical model that depicts how circadian variables are influenced by light exposure, feedback loops involving REV-ERB and ROR proteins within the clock mechanism, as well as how REV-ERB, ROR, and BMAL1-CLOCK affect the DAergic system. The predictions made by this mathematical model align with multiple experimental observations suggesting that the influence of REV-ERB, ROR, and BMAL1-CLOCK is sufficient for explaining circadian oscillations observed in DAergic variable levels. Furthermore, it can also predict circadian fluctuations in extracellular DA levels along with homovanillic acid (Kim and Reed, 2021).

In summary, changes in clock genes are closely related to DA synthesis. Loss of BMAL1 causes oxidative stress and synaptic loss in the mouse brain, and can aggravate DAergic neurodegeneration, the cellular autonomic role in the survival of DAergic neurons. And loss of DAergic neurons was noted in SNpc, but not in the hippocampus or other regions of the BMAL1-KO mice. Disruption of the clock gene Per interferes with the development of DA neurons in PAM clusters. REV-ERBα competes with NURR1 to directly inhibit TH expression and restrict DA synthesis, and inhibition of REV-ERBα can achieve anti-depression and anti-anxiety effects (Table 1).

**TABLE 1:** Changes in clock genes cause dopamine loss.

Clock gene	Model	Result	References
BMAL1	BMAL1-KO mice	BMAL1 caused spontaneous loss of TH dopaminergic neurons in the SNpc.	Kanan et al., 2024
PER	Drosophila	Disruption of circadian clocks interferes with the development of DA neurons in the PAM cluster and accelerates their age-dependent degeneration.	Majcin Dorcikova et al., 2023
REV-ERBα	6-OHDA-injected mice	REV-ERBα antagonist SR8278 exerted antidepressant and anxiolytic effects in a circadian time-dependent manner	Kim et al., 2022
REV-ERBα	6-OHDA-injected mice	NR1D1 deficiency significantly exacerbated 6-OHDA-induced motor deficits as well as DAergic neuronal loss in the vertebral midbrain.	Kim et al., 2018
BMAL1-CLOCK, REV-ERBs, RORs, BC and BMAL1	Mathematical model	Decoupling between the interlocked loops of a clock circuit or quasi-periodic clock behavior caused by inconsistencies with light-dark cycles can disrupt the daily rhythm of a DA.	Kim and Witelski, 2022
REV-ERB, ROR, and BMAL1-CLOCK	Mathematical model	The mechanisms by which REV-ERB, ROR, and BMAL1-CLOCK affect the DA system are sufficient to explain the circadian oscillations observed in dopaminergic variables.	Kim and Reed, 2021
light/dark (LD) ratios	MPTP-monkey	NP transplantation promotes the restoration of circadian rhythm and has neuroprotective and restorative effects on DA cell pool (VTA and SN dorsal medial and ventral striatum) target cells.	Wianny et al., 2022

### 2.2 Circadian rhythm disturbance mediated microglial activation

The research discovered that PD initiates with a process of neuroinflammation. In 2013, a study successfully identified the characteristics of neuroinflammation in a mouse model of PD prodrome, where α-synuclein (α-syn) overexpression was observed. This finding suggests that early inflammation could potentially lead to progressive lesions in the striatum-substantia nigra region. Furthermore, the team demonstrated the presence of sustained microglia-mediated neuroinflammation prior to any damage occurring in the striatum-substantia nigra circuit (Pradhan and Andreasson, 2013). Glial cells possess a circadian clock responsible for regulating their functions, ensuring brain development and maintaining homeostasis (Rojo et al., 2022). Recent evidence indicates that this circadian clock within microglia is involved in governing various physiological processes such as cytokine release, phagocytosis, and providing nutritional and metabolic support (Honzlová et al., 2023). The regulation of microglia’s circadian rhythm can significantly impact their immune response, phagocytosis function, metabolism, etc., all playing crucial roles in neurodegenerative diseases. Any disruption to this clock mechanism may have profound effects on multiple aspects related to PD, particularly concerning neuroinflammation and α-syn processes (Kou et al., 2024).

Nakazato et al. employed polymerase chain reaction for the initial detection of CLOCK genes in primary microglia and BV2 cell lines, encompassing BMAL1, CLOCK, PER1-3, CRY1-2, Dec1-2 and Npas2 (Nakazato et al., 2011). Subsequently, Hayashi et al. confirmed the presence of a clock gene in microglia and unveiled distinct circadian patterns of clock gene expression; for instance, PER1 and PER2 exhibited peak levels at zeitgeber time ZT14, REV-ERBα peaked at ZT18, while BMAL1 reached its maximum expression at ZT2 (Hayashi et al., 2013). Recent investigations have suggested that circadian oscillations of microglia clock genes form the basis for diurnal fluctuations in microglia physiological functions (Fonken et al., 2015).

In mice with MPTP-induced symptoms, the inactivation of BMAL1 led to notable impairments in motor function and a decrease in dopamine neurons and neurotransmitters in the SNpc. In comparison to MPTP-treated mice with intact BMAL1 (BMAL1+/+), those without BMAL1 (BMAL1–/–) exhibited significantly elevated expression of IBA1 and increased activation of microglia in the striatum. The absence of BMAL1 enhanced the activation of the NF-κB pathway induced by LPS through an increase in reactive oxygen species production. Following MPTP induction, BMAL1-deficient mice displayed reduced rotation time or calorie consumption, as well as decreased food and water intake, indicating that the loss of BMAL1 may exacerbate disruptions in circadian rhythms and neuroinflammation mediated by microglia (Liu et al., 2020). Other studies have similarly concluded that the depletion of BMAL1 triggers hyperplasia-associated activation of microglia (Kanan et al., 2024).

To investigate the potential role of the clock in BV2 microglia lines, we quantified the expression levels of PER2 and BMAL1 genes in resting BV2 microglia. Our analysis revealed significant oscillations observed through ECHO, with periods and phases corresponding to those observed at the transcriptional level for these genes. The expression patterns of clock genes PER2 and BMAL1 exhibited daily oscillations in BV2 microglia. Interestingly, exposure to LPS was found to inhibit the oscillation of clock genes PER2 and BMAL1 in BV2 microglia. Furthermore, under pro-inflammatory activation, the clock lost its rhythm while it maintained its rhythm under anti-inflammatory activation (Muthukumarasamy et al., 2023).

There exists a correlation between the circadian rhythm gene REV-ERBα and neuroinflammation in individuals with PD, as the absence of REV-ERBα intensifies the degeneration of dopamine-producing neurons induced by 6-OHDA, while also being linked to an increase in microglia proliferation within the substantia nigra (SN) (Kim et al., 2018). It was observed that the abnormal diurnal-day REV-ERBα rhythm in the SN of PD mice induced by MPTP could be detected by examining the mRNA level of REV-ERBα at different time points within 24 hours. Additionally, Iba1 immunofluorescence staining revealed the activation of microglia-mediated neuroinflammation in the SN of MPTP-induced PD mice. To investigate further, BV2 cells were pre-treated with varying concentrations (2uM, 5uM, 10uM) of SR9009, a REV-ERBα agonist, for one hour before being exposed to MPP. The results showed that SR9009 effectively inhibited the activation of NF-κB and NLRP3 inflammasome pathways. It is suggested that REV-ERBα may alleviate microglia activation induced by 1-methyl-4-phenylpyridine (MPP) through modulation of the NF-κB pathway and promote their transformation from an inflammatory M1 type to an anti-inflammatory M2 type. These findings indicate its potential role in suppressing inflammation and improving PD (Kou et al., 2022).

RORα, a crucial nuclear receptor involved in circadian rhythms, plays a regulatory role in the immune response. Melatonin (MLT), which could potentially act as an endogenous ligand for RORα, has been found to have immunoregulatory effects (He et al., 2016). In an experimental model using BV2 cells, it was observed that treatment with MPP ion downregulated the expression of RORα. This deficiency of RORα led to an enhanced proinflammatory phenotype and worsened neuroinflammation in microglia. However, MLT administration upregulated RORα expression and mitigated inflammation by reducing the gene expression associated with proinflammatory and damage-associated molecular patterns phenotypes in MPP-treated BV2 cells. In MPTP-induced mouse models, RORα levels were reduced in midbrain and MLT treatment (20 mg/kg/ day intraperitoneally for 7 days) significantly increased RORα levels, reduced inflammation and increased the anti-inflammatory M2-like phenotype in microglia (Li et al., 2022) (Table 2).

**TABLE 2:** Changes in clock genes lead to activation of microglia.

Clock gene	Model	Result	References
BMAL1	MPTP-treated mice	Loss of BMAL1 exacerbates microglia-induced neuroinflammation, loss of dopaminergic neurons, and motor function	Liu et al., 2020
PER2, BMAL1	BV2 mouse microglial cell	NOX2 inhibition enables retention of the circadian clock in BV2 microglia.	Muthukumarasamy et al., 2023
REV-ERBα	MPTP-treated mice and BV2 cell	REV-ERBα agonist SR9009 partially reversed MPTP induced microglial polarization, NLRP3 inflammasome activation and dopaminergic neurons loss in the nigrostriatal system.	Kou et al., 2022
RORα	MPP- treated BV2 cell	Significantly increases RORα levels and protects dopamine neurons, reduces inflammation and increases the anti-inflammatory M2-like phenotype in microglia.	Li et al., 2022

### 2.3 Disturbance of circadian rhythm leads to abnormal aggregation of α-syn

The pathogenesis of PD is closely linked to the abnormal accumulation of α-syn protein, which forms insoluble amyloid protein known as lewy bodies (Mehra et al., 2019). These aggregates are difficult to degrade once deposited in neurons, ultimately resulting in neuronal death (Surmeier et al., 2017). In individuals with PD, the presence of α-syn in specific brain regions has been strongly associated with disruptions in circadian rhythm and sleep patterns (Hatano et al., 2024). PD patients experiencing sleep disorders exhibit higher levels of α-syn load, particularly within brain regions involved in sleep regulation (Yan et al., 2022). One potential mechanism underlying the reduced activity of SCN neurons due to excessive α-syn expression could be alterations in synaptic transmission (Sharma et al., 2020). α-syn serves as a presynaptic protein that modulates the release of synaptic vesicles, and its misexpression can disrupt normal synaptic communication (Forloni, 2023). Neurons within SCN neural networks utilize γ-aminobutyric acid (GABA) as a neurotransmitter, continuously receiving GABA signals from other neurons (Chong et al., 2021). The overexpression of α-syn, which affects synaptic transmission, may disturb the balance between enhanced inhibition within the SCN network and consequently lead to circadian symptoms observed in mice.

Synuclein alpha (SNCA) gene mutations have been linked to both dominant variants and sporadic cases of PD (Agliardi et al., 2021). The heightened immune response to SNCA in A53T mice compared to WT mice in the SCN may be attributed to the accumulation of mutant A53T SNCA. This antibody is capable of detecting both the endogenous and mutated forms. The missense mutation A53T SNCA is associated with autosomal dominant early-onset familial PD. To assess its impact on the circadian system, this study examined the spontaneous motor behavior of non-transgenic (TG) wild-type mice and TG mice overexpressing the mutant human A53T SNCA. The study evaluated their responses under entrainment, free running conditions, as well as experimental jet lag scenarios. Additionally, immunohistochemistry was used to analyze vesicular glutamate transporter 2 (VGLUT2) in SCN. While the free-running circadian rhythm and production of circadian rhythms remained unaffected in A53T mice, they exhibited an advanced phase angle of 2.65 ± 0.5 hours before lights out when entrained into a light-dark cycle. Furthermore, these mice displayed impaired re-entrainment after experiencing experimental jet lag. Lastly, there was a reduction observed in VGLUT2 immune response within the SCN of A53T mice indicating impairment within their circadian system (Pfeffer et al., 2018).

The SNCA A53T overexpressed transgenic mice (SNCA A53T mice) had abnormal circadian rhythm behavior, and BMAL1 was down-regulated in SNCA A53T mice compared with their age-matched litters. The results showed that overexpression of SNCA decreased the stability of BMAL1 mRNA, induced circadian rhythm disturbance and down-regulated BMAL1 expression by transfecting miR-155 inhibitors (Liu et al., 2023).

Sleep deprivation (SD) at 18 months of age was observed to significantly increase insoluble α-syn in the cytosol of DAergic neurons in SNpc of Lrrk2^G2019S^ transgenic (TG) mice. These findings support the aggravating effect of SD on PD change. The study also found that the protein levels of the CLOCK gene changed most significantly after SD exposure in an age-dependent manner. There were significant differences in CLOCK proteins between SD and non-SD mice at 12 months of age (Liu et al., 2022) (Table 3).

**TABLE 3:** Changes in clock genes lead to abnormal aggregation of α-syn.

Clock gene	Model	Result	References
BMAL1	SNCA A53T-overexpressing transgenic mice	Overexpression of SNCA decreased the stability of BMAL1 mRNA, induced circadian rhythm disturbance and down-regulated BMAL1 expression.	Liu et al., 2023
CLOCK	Lrrk2^G2019S^transgenic mice	Sleep deprivation increased alpha-synuclein levels in the Lrrk2^G2019S^ transgenic mice.	Liu et al., 2022
Light/dark (LD) cycle	SNCAA53T-overexpressing transgenic mice	The SNCA immune response in the SCN of A53T mice was significantly increased, and the circadian rhythm system was impaired.	Pfeffer et al., 2018

## 3 Clinical manifestation perspective: the influence of circadian rhythm disturbance on PD

**FIGURE 2:**
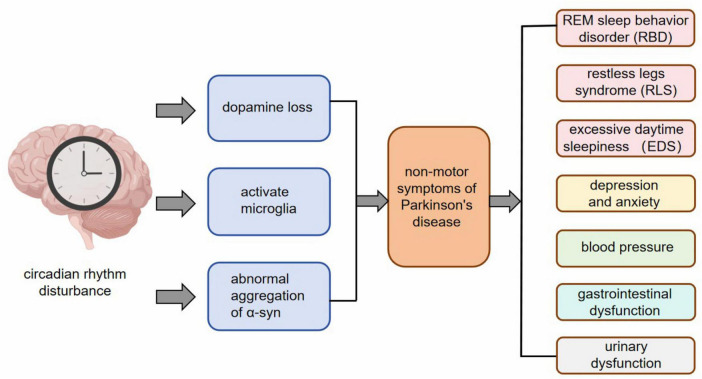
After circadian rhythm disturbance, pathological changes such as dopamine loss, microglia activation, and abnormal aggregation of α-syn appear at the cellular and molecular level, and then RBD, RLS, EDS, anxiety and depression, changes in blood pressure, gastrointestinal dysfunction, urinary dysfunction and other non-motor symptoms appear.

The non-motor manifestations of PD manifest at an early stage and can occur up to two decades prior to the official diagnosis of PD syndrome (Pont-Sunyer et al., 2015). According to the Movement Disorders Association, the non-movement symptoms associated with PD include REM sleep behavior disorder (RBD), abnormal findings in dopaminergic positron emission tomography, olfactory dysfunction, gastrointestinal issues such as constipation, excessive daytime sleepiness (EDS), anxiety-related depression, symptomatic hypotension, urinary problems, among others (Berg et al., 2015). These aforementioned non-motor symptoms have demonstrated a strong predictive value for the development of PD (Xie et al., 2019). It has been observed that nearly all non-motor symptoms experienced by individuals with PD are linked to disruptions in their circadian rhythm (Tarianyk et al., 2021). Recent studies provide mounting evidence suggesting that circadian dysrhythmia may be responsible for these non-motor symptoms observed in individuals with PD (Figure 2).

### 3.1 Rapid eye movement sleep behavior disorder

The sleep-wake cycle, regulated by the hypothalamus and reticular structure, is a well-known circadian rhythm that plays a crucial role in our daily lives. While the prevalence of rapid eye movement sleep behavior disorder (RBD) in the general population is less than 0.05% (Ohayon and Schenck, 2010), it occurs in up to 47% of individuals with PD (Högl et al., 2018). Among various sleep disorders, RBD can serve as an early indication of PD and heteromorphic sleep. It is characterized by abnormal behaviors and loss of muscle tone during REM sleep, such as vocalization, convulsions, and motor movements associated with dreams (Hu, 2020). In PD patients, there is an increase in REM sleep duration at night but no significant change in REM frequency compared to those without RBD. This suggests alterations in the circadian rhythm system related to RBD among PD patients (Feuerstein and Amara, 2023). The risk of developing neurodegenerative diseases within 14 years after experiencing RBD exceeds 90% (Iranzo et al., 2014), making it a valuable precursor marker for neurodegeneration, particularly synuclein diseases. Among all potential markers for predicting PD development, RBD demonstrates the highest predictive power and specificity (Dodet, 2023).

### 3.2 Restless legs syndrome

Restless legs syndrome (RLS), a sleep-wake disorder often coexisting with other conditions, has been observed in 3.9%–14.3% of the general population (Ohayon et al., 2012) and up to 16.3% of individuals with PD (Moccia et al., 2016). Individuals with RLS typically experience leg movement symptoms accompanied by sensory changes, which may worsen or improve during rest and physical activity respectively, while usually worsening at night (Gossard et al., 2021). The diagnosis of RLS requires the presence of symptoms exhibiting circadian rhythm fluctuations (Tang et al., 2023). Previous studies have suggested a potential link between the circadian rhythm variation of RLS and MLT secretion patterns (Hu et al., 2023). Interestingly, it has been noted that patients with RLS who consume melatonin supplements before bedtime often report more severe RLS symptoms, whereas exposure to bright light can reduce both MLT secretion and motor symptoms associated with RLS (Holder and Narula, 2022).

### 3.3 Excessive daytime sleepiness

MLT, a marker of the body’s internal circadian rhythm, is closely linked to excessive daytime sleepiness (EDS) in individuals. Research indicates that the prevalence of PD patients experiencing EDS ranges from 15% to 76% (Mantovani et al., 2018). Several studies have observed that PD patients exhibit a diminished circadian rhythm in MLT secretion, with significantly lower amplitude and area under the 24-hour curve (AUC) of circulating MLT levels compared to the control group. Among PD participants, those with EDS (ESS score ≥ 10) demonstrated even lower MLT rhythm amplitude and 24-hour MLT AUC than their counterparts without excessive sleepiness (Videnovic et al., 2014). The disruption of circadian function may serve as the underlying cause for excessive sleepiness in PD patients. Additionally, a longitudinal analysis revealed that by year 3, approximately 14.6% of non-dysphagia patients developed dysphagia symptoms. Both univariate and multivariate regression models strongly associated EDS measured by ESS with the development of dysphagia at year three (Marano et al., 2020).

### 3.4 Depression and anxiety

Individuals diagnosed with PD often experience disrupted sleep-wake cycles and poor quality of sleep, which can lead to mood disorders such as depression and anxiety (Walker et al., 2020). Depression affects approximately 45% of PD patients, while anxiety accompanies around 50% of cases (Brown et al., 2011). Research suggests a connection between circadian rhythm disturbances in PD patients and the development of depression and anxiety (Anyan et al., 2017). The term “sunset syndrome” or “nocturnal delirium” is used to describe mood disorders that tend to peak in the afternoon or evening among individuals with PD (Bedrosian and Nelson, 2013). Dopaminergic neurons located in the midbrain’s VTA innervate the prefrontal cortex, which is associated with mental disorders observed in PD patients (Ott and Nieder, 2019). Previous studies on animal models have demonstrated that circadian dysregulation worsens dopaminergic neuron loss. Additionally, variations in genes related to circadian rhythms have been linked to depression among individuals with PD (Hua et al., 2012).

### 3.5 Blood pressure

PD patients often exhibit distinct alterations in their blood pressure circadian rhythm, with nighttime hypertension and post-meal hypotension being notable characteristics (Kanegusuku et al., 2017). The disparity between daytime and nighttime blood pressure levels is significantly reduced in PD patients compared to healthy control groups. This disruption or absence of the circadian rhythm of blood pressure in PD patients is referred to as nocturnal non-dip (Vallelonga et al., 2019). A study discovered that individuals with PD who have an unaltered circadian rhythm tend to experience more severe psychotic symptoms than those with a disrupted circadian rhythm. On average, non-dippers had higher BPRS scores and met the criteria for psychosis. There was a correlation between BPRS scores and the absence of a decrease, suggesting that patients who experienced reduced or inactive blood pressure at night were more likely to exhibit psychotic symptoms (Stuebner et al., 2015).

### 3.6 Gastrointestinal dysfunction

Gastrointestinal dysfunction in individuals with PD is also associated with disturbances in the body’s internal clock (Gálvez-Robleño et al., 2023). PD patients without gastrointestinal dysfunction have higher levels of MLT in their plasma compared to those with gastrointestinal dysfunction (Li et al., 2020). It has been observed that the gastrointestinal tract, which is the second largest producer of MLT after the pineal gland, may play a significant role as a barrier for MLT within the body (Taslidere et al., 2018). This suggests that there exists a correlation between gastrointestinal dysfunction and circadian dysregulation among PD patients.

### 3.7 Urinary dysfunction

The production of urine is influenced by the regulation of sodium and free water treatment according to circadian rhythms. A study involving 115 patients with PD found that 53% of men and 63% of women experienced nocturia alongside PD (Sakakibara et al., 2001). The production of urine follows a circadian rhythm, with less than 25% being produced during nighttime in a 24-hour period. This is believed to be controlled by the release of Arginine vasopressin (AVP) and MLT (Matthiesen et al., 1996). The hormone’s release throughout the day is regulated by increased levels in plasma from the pituitary gland and arginine vasopressin at night. Older individuals who reported nocturnal polyuria (NP) were observed to have disrupted diurnal response, resulting in limited reabsorption of free water and increased urination. Decreased secretion of AVP not only contributes to nocturnal polyuria but also nocturnal enuresis. Disruptions in circadian rhythms not only affect urine production but also bladder function. Recent research using an OAB rat model demonstrated improved detrusor overactivity after administration of MLT (Batla et al., 2016).

### 3.8 Facing the future: future research direction of circadian rhythm prevention of PD development

The consideration of circadian rhythms in PD is gaining widespread attention. Hence, exploring chronobiological processes and implementing chronotherapy techniques could have practical implications for preventing and treating PD. However, the current state of research and development in this area is still at an early stage. After analyzing relevant literature, we have identified certain existing issues in current studies and put forth corresponding measures to address them as follows:

(1) In comparison to the molecular and cellular level, there is a lack of research on the clinical manifestations of non-motor symptoms in PD caused by disturbances in circadian rhythm. Existing studies briefly mention the impact of circadian rhythm disruption on specific non-motor symptoms, but fail to delve deeper into this topic. For instance, while some studies touch upon the influence of circadian clock genes on sleep disorders, they do not thoroughly distinguish between different types such as RBD, RLS, and EDS. Given that RBD serves as a highly specific indicator for predicting PD progression, we believe that conducting focused investigations on individual types would yield more valuable insights than general studies. Future research should aim to comprehensively explore various types of sleep disorders within the context of circadian rhythm-related clock genes throughout a 24-hour cycle.
(2) The circadian system has a widespread impact on physiological systems, potentially influencing PD through various processes. For instance, there is a hypothesis that PD may stem from the gut (Gershanik, 2018), and since the gut microbiome is strongly regulated by circadian rhythm, both circadian rhythm and gut microbiome are considered significant pathogenic factors in PD. However, much remains unknown about the alterations in gut microbiome following circadian disruption and the bidirectional interaction between circadian clock genes, gut microbiome, and gut function. Therefore, future research focusing on the interplay between circadian rhythm and gut microbiome holds great importance.
(3) Clinical trials should be systematically aligned with animal studies on PD at the preclinical stage. Animal models offer significant potential to enrich and supplement clinical research, but their utilization must be carefully evaluated in study design. Instead of conducting a single observation in a sole model, it is advisable to employ multiple models to identify the most pertinent commonalities that accurately represent the clinical situation of PD. When using TG mouse models, one must take into account the background strain, while also recognizing that wild-type animals may exhibit significant differences in circadian rhythm functions. Preclinical animal models for PD have the capacity to unveil the intricate connections between circadian rhythm functions, dopamine loss, neuroinflammation, abnormal accumulation of α-syn and other neurotoxins, as well as motor and non-motor symptoms of PD.
(4) Utilizing time-based therapy and light-based treatment to restore regular cellular functions may also aid in decelerating the advancement of neurodegenerative conditions by enhancing the identification and removal of misfolded proteins. Modifying the activation ratio of photoreceptors, which mirrors the retinal abnormalities identified in SCN and PD, could present prospects for formulating mechanism-oriented light therapy protocols, such as customizing the range of light therapy according to each individual’s needs. Depending on the significance of drug timing, these targeted light therapy protocols might require adjustments to complement melatonin or other medications for a synergistic therapeutic approach.
(5) At present, current research is placing emphasis on the convergence of interdisciplinary aspects, such as diverse mathematical models and diagnostic models. Some investigations have formulated diagnostic models for PD by utilizing circadian gene rhythms, demonstrating robust diagnostic effects (Tianjiao Wang et al., 2024). Nevertheless, there are certain constraints within the study. Firstly, the sample size is relatively limited, potentially impacting the external validity and reliability of the diagnostic model. Secondly, the study solely concentrated on investigating disrupted circadian gene rhythms in PD without taking into account the influence of other PD biomarkers on circadian gene rhythms, which could moderately affect the model’s accuracy. In future studies, it will be essential to utilize larger datasets to refine the model.
(6) Disruption of the circadian rhythm not only impacts non-motor symptoms but also demonstrates its influence on the seasonal variation of these symptoms. There is a seasonal disparity in the overall NMSS score, with the highest score occurring in the first quarter (winter) and the lowest score in the third quarter (summer). Seasonal differences are observed in NMSS domain 1 (cardiovascular symptoms), domain 4 (perceptual problems), and domain 9 (miscellaneous symptoms). Non-motor symptoms of PD patients fluctuate throughout the year, with exacerbation during winter compared to summer, largely due to the influence of the biological clock located in the hypothalamic SCN (van Wamelen et al., 2019). The circadian rhythm impacts all aspects of this disease, thus suggesting that future research should have a broader focus.

## 4 Discussion

Following the reception of the Nobel Prize in 2017, there has been a growing interest in circadian rhythm among the general public. This article takes a comprehensive approach to elucidate how circadian rhythm disorder contributes to PD by examining cellular molecular mechanisms and clinical symptoms horizontally, while also exploring the potential of circadian rhythm in preventing and slowing down the progression of PD through current research and future directions vertically. The study of circadian rhythm’s role in PD is still at an early stage. There exists a complex interplay between the circadian clock gene system, circadian rhythm, and the pathophysiology of PD. Given that non-motor symptoms can manifest up to 20 years before an official diagnosis of PD syndrome, it is crucial to comprehend how circadian rhythm disorder affects cellular molecular mechanisms and non-motor symptoms. The regulation of clock genes’ activity offers promising prospects for treating this condition.
